# Comment on “Shiga-Toxin Producing *Escherichia coli* in Brazil: A Systematic Review. *Microorganisms* 2019, *7*, 137”

**DOI:** 10.3390/microorganisms7100417

**Published:** 2019-10-03

**Authors:** Beatriz E. C. Guth

**Affiliations:** Department of Microbiology, Immunology, Parasitology, Escola Paulista de Medicina, Universidade Federal de São Paulo, São Paulo 04023-062, Brazil; bec.guth@unifesp.br

A recent article by Castro et al. describes a systematic review of Shiga-toxin producing *Escherichia coli* (STEC) in Brazil. In this article, the authors claim that the prevalence and distribution of STEC serogroups in Brazil remain unclear. However, this affirmation should not be considered true; as otherwise all the conducted surveys would not have been possible. In addition, some statements and conclusions are incorrect, and false hypotheses have been suggested.

Castro et al. wrote a systematic review on STEC in Brazil by selecting papers from online databases (according to Prisma guidelines), and limiting their search for articles published between January 2000 and December 2018 [1]. Collecting data produced by others is not an easy task; and the authors should be very cautious when making statements and concluding remarks that are not supported by the selection of references and parameters used.

Indeed, STEC infections in Brazil are mainly represented by sporadic cases of diseases, and STEC outbreaks are uncommon. However, this does not mean that “... *no STEC outbreak case in Brazil has been reported*”, as was mentioned by the authors in their abstract and conclusions. In fact, looking at the genetic diversity of O157:H7 strains by PFGE, Vaz et al. [2] reported the first occurrence of an O157:H7 STEC outbreak in Brazil. This information was also reinforced in another publication by Bastos et al. [3], in which another O157:H7 STEC strain that was isolated from a hemolytic uremic syndrome (HUS) case in 2005 in São Paulo, Brazil—presented the same PFGE profile of the outbreak O157:H7 human strains previously described. Altogether, these data suggest the presence and maintenance of a specific O157 clone responsible for human infections in our settings.

Moreover, in an attempt to explain that there were no reports of outbreaks involving STEC, Castro et al. [1] stated two untrue hypotheses “(i): *Disease outbreaks are not recorded by lack of centralized reporting system or (ii) disease outbreaks are not being recognized as there are no surveillance systems for STEC*”. In fact, in São Paulo, the active surveillance of HUS, bloody diarrhea, and *E. coli* are part of the active surveillance system for foodborne diseases (DTA), the Monitoring of Acute Diarrheal Diseases (MDDA), and the surveillance of outbreaks of DTA [4]. Furthermore, due to the importance of STEC infections in public health, HUS surveillance is compulsorily notified at a national level, and surveillance is based on a sentinel system [5]. Possible explanations on the low incidence of STEC outbreaks in Brazil is beyond the revision made by Castro et al. [1], and has been explored in other publications [6,7].

Another important point of disagreement is the analysis based on the most frequent STEC serotypes isolated from animals, food, and humans, represented and summarized in Figure 2 of the aforementioned article [1]. The authors concluded that “*O157:H7 serotype had the highest occurrence rates in animal, food and humans in Brazil, and that this higher prevalence might be related to its ease detection*” [1]. Unfortunately, these conclusions cannot be confirmed by the data and references presented in Tables 1–3 [1]. Although serotype O157:H7 has been identified in different animal species, its frequency is very low (0.9% to 1.9%) compared to other non-O157 STEC serotypes [8,9,10,11]. Indeed, in the study of Oliveira et al. [11], serotype O157:H7 was recovered only from one beef cattle. The authors tried to justify this low incidence and the failure to recover it from dairy cattle by the lack of enrichment use, as well as more sensitive and specific methods. On the other hand, O157:H7 STEC has not been detected from diarrheic dogs [12], as described in Table 1 [1]. Caution in listing the prevalence of STEC isolated in food produced in Brazil, as shown in Table 2 [1], also needs to be taken, as in cheese samples the paper referenced [13] just described the isolation of *E. coli* O157:H7, production of Shiga toxin or detection of *stx* genes that classify a strain as STEC was not performed. The same comment is valid for the isolation of serogroups O125, O111, O55, and O119 from raw milk that as described in the referenced paper [14] did not carry *stx* genes. Considering the prevalence of STEC in humans [1], another relevant correction to be made regarding the O157:H7 serotype, which is the most frequent serotype associated with more severe infections, such as HUS and hemorrhagic colitis. However, if we consider human infections as a whole, non-O157 serotypes (such as O111:H8, O103:H2, O118:H16, and several others), are most frequently identified [15,16]. Although Castro et al. [1] mentioned that O118:H16 serotype was only detected in human clinical cases, this serotype was also isolated and identified from fecal samples of cattle as described by Leomil et al. [17].

Lastly, Castro et al. [1] claimed that the prevalence and distribution of STEC serogroups in Brazil remain unclear. This statement cannot be considered true: Otherwise, all the surveys conducted by the authors, and shown in Tables 1–3, would not have been possible. However, considering that the authors sought to perform a systematic review of STEC, it was expected that a careful analysis of the data in the different selected studies had been performed. Otherwise, one can generalize information that does not actually match the referenced data.

In conclusion, the comments presented herein are appropriate and important, in light of our knowledge about the epidemiology of STEC infections in Brazil.
